# Modal-Transition-Induced Valleys of *K*^2^ in Piezoelectric Bilayer Laterally Vibrating Resonators

**DOI:** 10.3390/mi14051022

**Published:** 2023-05-10

**Authors:** Zihao Xie, Jiabao Sun, Jin Xie

**Affiliations:** 1 State Key Laboratory of Fluid Power and Mechatronic Systems, Zhejiang University, Hangzhou 310027, China; xiezihao@zju.edu.cn; 2 Micro-Nano Fabrication Center, Zhejiang University, Hangzhou 310027, China; jbsun@zju.edu.cn

**Keywords:** electromechanical coupling factor, finite element analysis, laterally vibrating resonators, modal transition, piezoelectric bilayer resonators, thermal compensation

## Abstract

Piezoelectric Laterally Vibrating Resonators (LVRs) have attracted significant attention as a potential technology for next-generation wafer-level multi-band filters. Piezoelectric bilayer structures such as Thin-film Piezoelectric-on-Silicon (TPoS) LVRs which aim to increase the quality factor (*Q*) or aluminum nitride and silicon dioxide (AlN/SiO_2_) composite membrane for thermal compensation have been proposed. However, limited studies have investigated the detailed behaviors of the electromechanical coupling factor (*K*^2^) of these piezoelectric bilayer LVRs. Herein, AlN/Si bilayer LVRs are selected as an example, we observed notable degenerative valleys in *K*^2^ at specific normalized thicknesses using two-dimensional finite element analysis (FEA), which has not been reported in the previous studies of bilayer LVRs. Moreover, the bilayer LVRs should be designed away from the valleys to minimize the reduction in *K*^2^. Modal-transition-induced mismatch between electric and strain fields of AlN/Si bilayer LVRs are investigated to interpret the valleys from energy considerations. Furthermore, the impact of various factors, including electrode configurations, AlN/Si thickness ratios, the Number of Interdigitated Electrode (IDT) Fingers (NFs), and IDT Duty Factors (DFs), on the observed valleys and *K*^2^ are analyzed. These results can provide guidance for the designs of piezoelectric LVRs with bilayer structure, especially for LVRs with a moderate *K*^2^ and low thickness ratio.

## 1. Introduction

With the maturity of piezoelectric thin film fabrication processes and the increasing demand for miniaturized, low-power, multi-band RF devices, piezoelectric acoustic resonators such as Surface Acoustic Wave (SAW) resonators and Bulk Acoustic Wave (BAW) resonators have gained widespread commercial applications. The SAW resonators initially dominated the filter market due to their low cost and high tolerance for fabrication errors. However, the relatively low phase velocity of Rayleigh waves limited their applications in the super high frequency (SHF) range, and the SAW modes do not fully utilize the piezoelectric transduction capability [1,2]. The BAW resonators, represented by Film Bulk Acoustic Resonators (FBARs), have a high phase velocity (11,400 m/s for aluminum nitride (AlN) ) [3] and are fully compatible with IC technology, making up for the limitations of the SAW resonators. Additionally, the FBARs exhibit a high quality factor and extremely low insertion loss [4,5]. However, the FBARs operate in thickness extensional mode, so the resonant frequency is determined by the thickness of the piezoelectric plate, making it impossible to integrate a multi-band filter onto a single chip through lithography technology. On the other hand, Laterally Vibrating Resonators (LVRs) operate in the lateral strain resonant mode induced by the electric field in the thickness direction, and the resonant frequency is mainly determined by the in-plane dimensions, enabling wafer-level multi-band filters [6,7,8,9]. One major disadvantage of LVRs compared to FBARs is the moderate electromechanical coupling coefficient ($k_{t}^{2}$) due to the limited piezoelectric coefficient $d_{31}$ of piezoelectric material, such as AlN. Cross-sectional Lamé mode resonators combining $d_{31}$ and $d_{33}$ have been proposed to enhance the $k_{t}^{2}$ of LVRs up to 7% [10].

Common LVRs can be broadly classified into two categories based on the presence or absence of a structural layer at the bottom: pure piezoelectric (PP) layer LVRs and bilayer LVRs [11,12]. The PP LVRs utilize IDT for exciting the lowest-order symmetric (S_0_) Lamb mode, also known as Lamb wave resonators. The S_0_ mode is particularly favorable due to its high phase velocity (exceeding 9800 m/s for AlN) and weak phase velocity dispersion [13]. The structural layer of the bilayer LVRs is introduced for performance enhancement. For example, AlN on silicon dioxide (SiO_2_) or lithium niobate (LiNbO_3_) on SiO_2_ LVRs are proposed as a solution for temperature compensation [14,15]. Furthermore, the bottom layer of Thin-Film Piezoelectric-on-Silicon (TPoS) LVRs is typically made of a semiconductor or insulating material with a high intrinsic *Q* value, such as single-crystal silicon, to improve the *Q* value of LVRs [16,17,18]. However, the bilayer LVRs do not exhibit purely symmetric Lamb modes due to the asymmetry of the materials in the thickness direction and instead operate in the lowest-order quasi-symmetric (QS_0_) mode [19,20].

Incorporating a structural layer leads to a decrease in electromechanical coupling factor (*K*^2^) due to the decrease in motional capacitance and static capacitance maintenance, which limits the maximum bandwidth of the filters [1,17]. To minimize the reduction in *K*^2^, it is essential to carefully design the structural parameters of the TPoS LVRs and the electrode configurations. Vladimir et al. utilized Adler’s approach [21] to investigate the *K*^2^ of AlN/Si and ZnO/Si bilayer resonators under different order resonant modes and normalized thicknesses [22]; Laidoudi et al. employed the same approach to study the *K*^2^ of ZnO/SiC bilayer resonator under various rotation angles and normalized thicknesses [23]. However, there is a slight difference between the *K*^2^ obtained by Adler’s approach and the $k_{t}^{2}$ for LVRs as the normalized thickness increase [2]. In addition, Adler’s approach cannot distinguish between the electrode configurations of IDT-floating and IDT-grounded [24]. Zou et al. used two-dimensional finite element analysis (FEA) to study the influence of electrode configurations and normalized AlN thicknesses on the PP LVRs, with all simulations based on the assumption of periodic boundary conditions (PBCs) [2]. The results calculated from PBCs are sufficient for LVRs with a high number of interdigitated electrode (IDT) Fingers (NFs) but are not accurate enough to represent the *K*^2^ of bilayer LVRs with a low NFs.

In this paper, the Quasi-Lamb (QL) modes and *K*^2^ of the AlN/Si bilayer plate are investigated first. Subsequently, the behavior of the QS_0_ mode and *K*^2^ of the AlN/Si bilayer LVRs are studied through two-dimensional FEA with COMSOL and compared to the results obtained through Adler’s approach. Significant degenerative valleys in *K*^2^ are observed at specific normalized thicknesses, which is interpreted in terms of modal transition and the energy definition of *K*^2^. Finally, the impact of various factors, including electrode configurations, AlN/Si thickness ratios, NF, and IDT duty factors (DFs) on *K*^2^ are analyzed.

## 2. QL Modes and *K*^2^ of the AlN/Si Bilayer Plate

**Figure 1:**
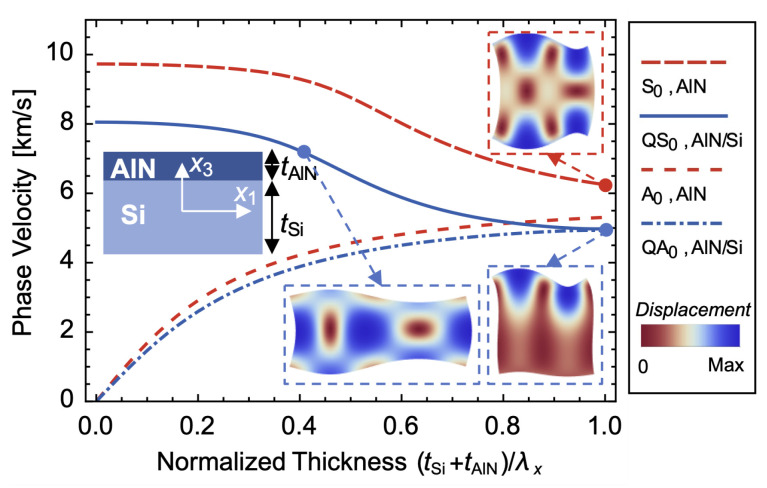
Phase velocity dispersion curves for both an AlN/Si bilayer plate and a pure AlN plate, and displacement shapes of both the S_0_ and QS_0_ branches depicted at normalized thicknesses of 0.4 and 1.0.

Lamb waves are elastic guided waves formed by the reflection of longitudinal waves and shear-vertical waves polarized in the sagittal plane at parallel boundaries in an infinite plate. Sinusoidal solutions of Lamb’s characteristic equations contain symmetric and antisymmetric modes, whose motion of particles is Symmetric (S) or Antisymmetric (A) about the midplane of the plate. However, S and A modes do not exist in the AlN/Si bilayer plate as shown in the inset of Figure 1, whose sinusoidal wave solutions are generally expressed as [25,26]

(1) $$\left\{\begin{array}{cc} u_{1}= & \cos\left(k_{1}x_{1}\right)\sum_{i=1}^{3}C_{i}\left(\beta_{1,i}\cos\left(k_{3,i}x_{3}\right)+\gamma_{1,i}\sin\left(k_{3,i}x_{3}\right)\right)\\ u_{3}= & \sin\left(k_{1}x_{1}\right)\sum_{i=1}^{3}C_{i}\left(\beta_{2,i}\sin\left(k_{3,i}x_{3}\right)+\gamma_{2,i}\cos\left(k_{3,i}x_{3}\right)\right)\\ \phi = & \sin\left(k_{1}x_{1}\right)\sum_{i=1}^{3}C_{i}\left(\beta_{3,i}\sin\left(k_{3,i}x_{3}\right)+\gamma_{3,i}\cos\left(k_{3,i}x_{3}\right)\right)\\ u_{1}^{\prime }= & \cos\left(k_{1}x_{1}\right)\sum_{i=1}^{3}C_{i}^{\prime }\left(\beta_{1,i}^{\prime }\cos\left(k_{3,i}^{\prime }x_{3}\right)+\gamma_{1,i}^{\prime }\sin\left(k_{3,i}^{\prime }x_{3}\right)\right)\\ u_{3}^{\prime }= & \sin\left(k_{1}x_{1}\right)\sum_{i=1}^{3}C_{i}^{\prime }\left(\beta_{2,i}^{\prime }\sin\left(k_{3,i}^{\prime }x_{3}\right)+\gamma_{2,i}^{\prime }\cos\left(k_{3,i}^{\prime }x_{3}\right)\right) \end{array}\right.$$

The solutions for the displacements and potential of the AlN (Si) layer are a linear combination of plane waves with wavenumbers $k_{1}$ in the direction $x_{1}$ and $k_{3,i}\;\left(k_{3,i}^{\prime }\right)$ in the $x_{3}$ direction, and the time-dependent harmonic term with an angular frequency of ω is omitted. The variables of $u_{1}\;\left(u_{1}^{\prime }\right)$, $u_{3}\;\left(u_{3}^{\prime }\right)$ and ϕ in the AlN (Si) layer denote the displacement in the direction, direction and potential, respectively. For a given lateral wavenumber $k_{1}$, vertical wavenumbers $k_{3,i}$, $k_{3,i}^{\prime }$ and the corresponding coefficient ratios $\beta_{1,i}:\gamma_{1,i}:\beta_{2,i}:\gamma_{2,i}:\beta_{3,i}:\gamma_{3,i}$ and $\beta_{1,i}^{\prime }:\gamma_{1,i}^{\prime }:\beta_{2,i}^{\prime }:\gamma_{2,i}^{\prime }$ are determined by setting the coefficient determinants of the stress equations of motion to zero. The coefficients $C_{i}$ and $C_{i}^{\prime }$ are then calculated using the constraints imposed by the free boundary conditions and stress continuity conditions [25].

Displacements and potential in (1) consist of symmetric and antisymmetric terms with coefficients $\beta_{j,i}\;\left(\beta_{j,i}^{\prime }\right)$ and $\gamma_{j,i}\;\left(\gamma_{j,i}^{\prime }\right)$, and these modes are referred to as QL modes. When $|\beta_{j,i}\left|\gg \right|\gamma_{j,i}|$ and $|\beta_{j,i}^{\prime }\left|\gg \right|\gamma_{j,i}^{\prime }|\;\left(i=1,\;2,\;3,\;j=1,\;2\right)$, the displacements are dominated by the symmetric components, and the corresponding QL mode is referred to as a quasi-symmetric (QS) mode. Conversely, when $|\beta_{j,i}\left|\ll \right|\gamma_{j,i}|$ and $|\beta_{j,i}^{\prime }\left|\ll \right|\gamma_{j,i}^{\prime }|\;\left(i=1,\;2,\;3,\;j=1,\;2\right)$, the displacements display predominantly antisymmetric behavior, and the QL mode is referred to as a quasi-antisymmetric (QA) mode.

The phase velocity $v_{p}=\omega /k_{x}$ dispersion curves for both an AlN/Si bilayer plate and a pure AlN plate are depicted in Figure 1. The material constants of AlN and Si are listed in Table 1 [27,28]. The thickness ratio of Si to AlN $t_{\mathrm{Si}}/t_{\mathrm{AlN}}$ is assumed to be 10 initially as an example. At a lower normalized thickness $\left(t_{\mathrm{Si}}+t_{\mathrm{AlN}}\right)/\lambda_{x}$, the phase velocity of the QS_0_ branch decreases from 9729 m/s for the S_0_ branch of AlN to 8051 m/s due to the lower phase velocity of Si. The displacement shape is nearly symmetric about the midplane when the normalized thickness equals 0.4, as demonstrated in Figure 1, and is mainly characterized by the lateral extensional displacement. As the normalized thickness increases, the phase velocities of QS_0_ and QA_0_ branches converge with that of the Rayleigh waves. At a normalized thickness of 1, the displacement shape of the QS_0_ mode resembles that of Rayleigh waves, and the symmetry almost disappears. On the other hand, the displacement of the S_0_ mode remains symmetric about the midplane.

**Figure 2:**
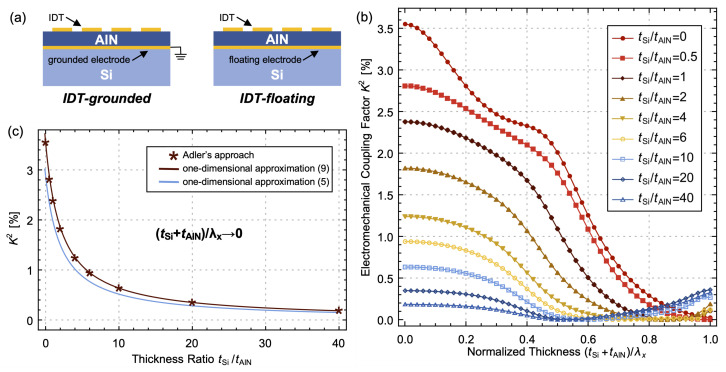
(**a**) Schematic diagrams of IDT-grounded and IDT-floating configurations; (**b**) *K*^2^ of the AlN/Si bilayer plate with normalized thickness from 0 to 1 at different thickness ratios; (**c**) comparison of *K*^2^ obtained through Adler’s approach and one-dimensional approximations (5) and (9).

The parameter *K*^2^ can effectively quantify the energy conversion efficiency for QL modes. Its value mainly depends on the piezoelectric coefficients, the ratio of plate thickness, and the electrode configurations. Two common configurations of electrode for the AlN/Si bilayer plate are illustrated in Figure 2a. Both configurations employ a top IDT covering the AlN layer, while the bottom electrodes are either grounded (IDT-grounded) or floating (IDT-floating). The *K*^2^ can be estimated through Adler’s approach by measuring the relative difference in phase velocity under electrically open and shorted surface boundary conditions [21]:

(2) $$K^{2}=\frac{v_{o}^{2}-v_{s}^{2}}{v_{s}^{2}}$$

where $v_{o}$ and $v_{s}$ represent the phase velocity under the open and shorted boundary conditions, respectively. These two configurations of electrode have the same $v_{o}$ and $v_{s}$, resulting in the same *K*^2^. Figure 2b depicts the calculated *K*^2^ for the AlN/Si bilayer plate with various thickness ratios from 0 to 40. The electrodes are assumed to be massless. The data presented in Figure 2b show a gradual decline in *K*^2^ as the thickness ratio increases, which will be discussed in detail later. Additionally, *K*^2^ displays a trend of initially decreasing, followed by an increase within the normalized thickness range from 0 to 1. The inflection point of this trend shifts toward lower normalized thickness with an increasing ratio of thickness. This phenomenon can be attributed to the transformation of the displacement shape from a lateral extensional mode to a Rayleigh-waves-like mode as the normalized thickness increases and the proximity of the QS_0_ and QA_0_ branches as the thickness ratio increases.

The *K*^2^ can be derived from the energy considerations [29,30]

(3) $$K^{2}=\frac{W_{\mathrm{mutual}}^{2}}{W_{\mathrm{mech}}W_{\mathrm{elec}}}=\frac{{\left(\int_{V}-\frac{1}{2}e_{ki}E_{k}S_{i}dV\right)}^{2}}{\int_{V}\frac{1}{2}\varepsilon_{ij}E_{i}E_{j}dV\times \int_{V}\frac{1}{2}c_{ij}S_{i}S_{j}dV}$$

where the mutual energy $W_{\mathrm{mutual}}$ represents the coupling between the electrical and mechanical domains through the piezoelectric coupling coefficients $e_{ki}$, $W_{\mathrm{mech}}$ and $W_{\mathrm{elec}}$ characterize the energy stored in the mechanical and electrical domains. $E_{i}$ and $S_{i}$ denote the electric fields and strains, respectively, while $e_{ki}$, $\varepsilon_{ij}$ and $c_{ij}$ are piezoelectric constants, dielectric constants and stiffness constants. For AlN with hexagonal crystal symmetry, under the plane strain assumption, $S_{2}=S_{4}=S_{6}=E_{2}=0$, *K*^2^ can be expressed as

(4) $$K^{2}=\frac{{\left(\int_{S}\left(e_{31}E_{3}S_{1}+e_{33}E_{3}S_{3}+e_{15}E_{1}S_{5}\right)dS\right)}^{2}}{\int_{S}\left(\varepsilon_{11}E_{1}^{2}+\varepsilon_{33}E_{3}^{2}\right)dS\times \int_{S}\left[\begin{array}{ccc} S_{1} & S_{3} & S_{5} \end{array}\right]\left[\begin{array}{ccc} c_{11} & c_{13} & 0\\ c_{13} & c_{33} & 0\\ 0 & 0 & c_{44} \end{array}\right]\left[\begin{array}{c} S_{1}\\ S_{3}\\ S_{5} \end{array}\right]dS}$$

Furthermore, when the normalized thickness tends to zero, stresses $T_{3}=T_{5}=0$. For the QS_0_ mode and electrode configuration of IDT-floating, $S_{1}=A_{1}\sin\left(k_{1}x_{1}\right)$ and $E_{3}=\pm \Phi /t_{\mathrm{AlN}}$. Therefore, the one-dimensional approximation of the *K*^2^ for the AlN/Si bilayer plate at a lower normalized thickness is

(5) $$K^{2}=\frac{8}{\pi^{2}}\frac{{\tilde{e}}_{31}^{2}}{{\tilde{\varepsilon }}_{33}{\tilde{c}}_{11,\mathrm{AlN}}}\frac{t_{\mathrm{AlN}}{\tilde{c}}_{11,\mathrm{AlN}}}{t_{\mathrm{AlN}}{\tilde{c}}_{11,\mathrm{AlN}}+t_{\mathrm{Si}}{\tilde{c}}_{11,\mathrm{Si}}}$$

where

(6) $$\left\{\begin{array}{c} {\tilde{e}}_{31}=e_{31}-\frac{c_{13,\mathrm{AlN}}e_{33}}{c_{33,\mathrm{AlN}}},\;{\tilde{\varepsilon }}_{33}=\varepsilon_{33}+\frac{e_{33}^{2}}{c_{33,\mathrm{AlN}}}\\ {\tilde{c}}_{11,\mathrm{AlN}}=c_{11,\mathrm{AlN}}-\frac{c_{13,\mathrm{AlN}}^{2}}{c_{33,\mathrm{AlN}}},\;{\tilde{c}}_{11,\mathrm{AlN}}=c_{11,\mathrm{Si}}-\frac{c_{13,\mathrm{Si}}^{2}}{c_{33,\mathrm{Si}}} \end{array}\right.$$

The DF is assumed to be 1, and the vertical electric field permeates the entire AlN layer. In the case of IDT-grounded configuration, the electric field is present in only half of the AlN layer. Consequently, this induces half of the *K*^2^ value:

(7) $$K^{2}=\frac{4}{\pi^{2}}\frac{{\tilde{e}}_{31}^{2}}{{\tilde{\varepsilon }}_{33}{\tilde{c}}_{11,\mathrm{AlN}}}\frac{t_{\mathrm{AlN}}{\tilde{c}}_{11,\mathrm{AlN}}}{t_{\mathrm{AlN}}{\tilde{c}}_{11,\mathrm{AlN}}+t_{\mathrm{Si}}{\tilde{c}}_{11,\mathrm{Si}}}$$

The one-dimensional approximation demonstrates that a rise in normalized thickness results in a reduction in the *K*^2^ value. This approximation is compared to the results obtained through Adler’s approach when the normalized thickness approaches zero, as presented in Figure 2c. It is observed that the *K*^2^ value computed using Adler’s approach is marginally greater than that of the one-dimensional approximation for the IDT-floating configuration. In fact, when the surface of the AlN layer is either open or shortened, the vertical electric field matches the lateral strain, i.e., $E_{3}=-{\tilde{e}}_{31}/{\tilde{\varepsilon }}_{33}S_{1}$ or $E_{3}=0$, and the respective phase velocities are

(8) $$v_{o}=\sqrt{\frac{\left({\tilde{c}}_{11,\mathrm{AIN}}+{\tilde{e}}_{31}^{2}/{\tilde{\varepsilon }}_{33}\right)t_{\mathrm{AIN}}+{\tilde{c}}_{11,\mathrm{Si}}t_{\mathrm{Si}}}{\rho_{\mathrm{AIN}}t_{\mathrm{AIN}}+\rho_{\mathrm{Si}}t_{\mathrm{Si}}}},\;v_{s}=\sqrt{\frac{{\tilde{c}}_{11,\mathrm{AlN}}t_{\mathrm{AIN}}+{\tilde{c}}_{11,\mathrm{Si}}t_{\mathrm{Si}}}{\rho_{\mathrm{AlN}}t_{\mathrm{AlN}}+\rho_{\mathrm{Si}}t_{\mathrm{Si}}}}$$

Therefore, the *K*^2^ estimated through Adler’s approach at a lower normalized thickness is

(9) $$K^{2}=\frac{{\tilde{e}}_{31}^{2}}{{\tilde{\varepsilon }}_{33}{\tilde{c}}_{11,\mathrm{AlN}}}\frac{t_{\mathrm{AlN}}{\tilde{c}}_{11,\mathrm{AlN}}}{t_{\mathrm{AlN}}{\tilde{c}}_{11,\mathrm{AlN}}+t_{\mathrm{Si}}{\tilde{c}}_{11,\mathrm{Si}}}$$

The discrepancy in the coefficients between (5) and (9), $8/\pi^{2}$, stems from the mismatch between the excitation electric field and the strain field. This is because the uniform electric field in the lateral direction cannot match the sinusoidal strain. As the mismatch increases, Adler’s approach is less suitable for the analysis of QL waves, which will be demonstrated in the subsequent section. To obtain a more precise estimation of the *K*^2^ parameter, the Green’s function method can be employed [31]. Several different definitions of *K*^2^ mentioned in this article are summarized in Table 2.

## 3. *K*^2^ and Degenerative Valleys for AlN/Si Bilayer LVRs

Compared to the AlN/Si bilayer plate, AlN/Si bilayer LVRs have limited lateral dimensions. Due to the stress-free conditions on both sides of the resonator, the lateral dimension *L* of the resonator typically corresponds to an integer multiple of the half wavelength, $L=n\times \lambda_{x}/2$. When *n* is even, the resonant mode is antisymmetric to $x_{1}$, as shown in the mode shape of Figure 1 (n=2). Therefore, even-order modes can be expressed by the linear superposition of (1) with various wavenumbers $k_{1}$. Conversely, when *n* is odd, the resonant mode is symmetric to $x_{1}$, and it is necessary to exchange the antisymmetric and symmetric terms of $x_{1}$ in (1).

It is worth noting that, when calculating the dispersion curves for the AlN/Si bilayer plate, the two symmetric or antisymmetric standing wave solutions do not have a fundamental distinction but only present a phase difference due to the absence of boundary conditions in the direction of the plate. For AlN/Si bilayer LVRs, the even-order modes and odd-order modes are decoupled, but any two modes with the order of same parity are not decoupled.

**Figure 3:**
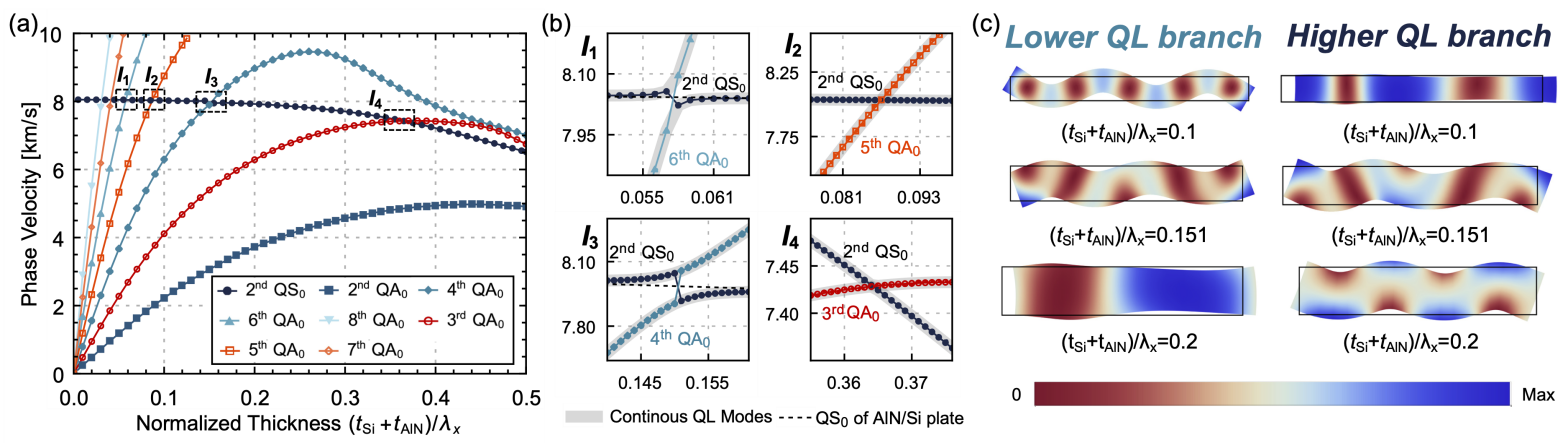
(**a**) Phase velocity dispersion curve, (**b**) magnified views and (**c**) resonant displacement modes of two QL branches of an AlN/Si bilayer LVR with a thickness ratio of 10, NF = 2 and DF = 0.8.

The phase velocity dispersion curves of an AlN/Si bilayer LVR with a thickness ratio of 10, NF = 2, and DF = 0.8 operating in the second QS_0_ mode are presented in Figure 3a, with magnified views of the relevant regions of interest depicted in Figure 3b. The coupling between all even-order QL modes results in the absence of intersections between any two even-order mode branches, and a modal transition is observed as they approach [32]. As shown in Figure 3b, near a normalized thickness of 0.151 (I_3_), the two QL branches approach and then diverge rapidly, with the upper branch evolving from the second QS_0_ mode to the fourth QA_0_ mode and the lower branch evolving from the fourth QA_0_ mode to the second QS_0_ mode. Figure 3c illustrates the resonant displacement modes of the two QL branches with normalized thicknesses around 0.151. It is observed that the two QL modes display typical bending and extensional displacement modes at the normalized thickness of 0.1, thus allowing for easy identification between the fourth QA_0_ mode and the second QS_0_ mode. When the normalized thickness increases to 0.151, the two QL branches approach, and the modes exhibit non-classical bending behaviors, making it difficult to categorize them as QS_0_ or QA_0_ modes. To maintain modal continuity, the mode with phase velocity close to the QS_0_ dispersion curve of the AlN/Si bilayer plate is labeled as the QS_0_ mode. As the normalized thickness increases further to 0.2, the two branches move away from each other and evolve into the second QS_0_ and fourth QA_0_ modes. Similarly, a modal transition is observed around the normalized thickness of 0.058 (I_1_) for the second QS_0_ mode and sixth QA_0_ mode. Due to the decoupling between even-order QL modes and odd-order QL modes, the second QS_0_ mode does not interact with odd-order QA_0_ mode branches, and there is an intersection between the second QS_0_ mode and the third (fifth) QA_0_ mode, as depicted in Figure 3b (I_2_, I_4_).

**Figure 4:**
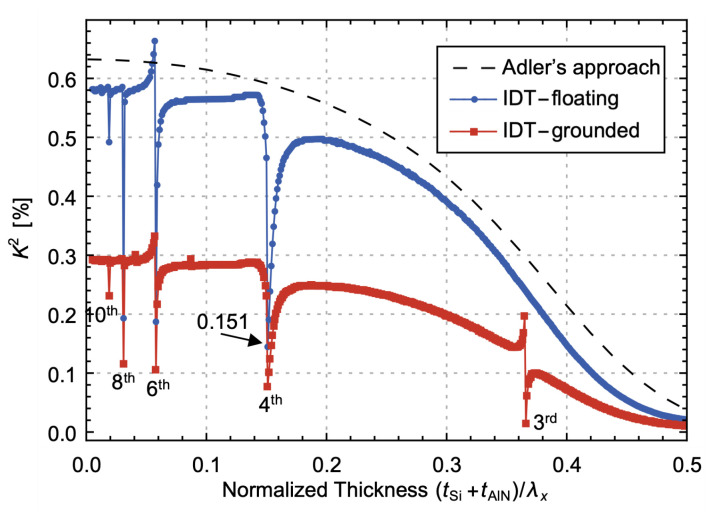
Simulated *K*^2^ of an AlN/Si bilayer LVR with electrode configurations of IDT-floating and IDT-grounded, with Adler’s approaching results of the AlN/Si bilayer plate for comparison. The thickness ratio is set to 10 and N = 2, DF = 0.8.

The calculation of *K*^2^ using an energy-based definition can be cumbersome in the analysis of resonators. Therefore, approximate definitions are commonly used [33]:

(10) $$K^{2}=\frac{f_{p}^{2}-f_{s}^{2}}{f_{s}^{2}}$$

where $f_{p}$ and $f_{s}$ are the parallel and series resonant frequencies, respectively. The $f_{p}$ and $f_{s}$ of the second QS_0_ mode are extracted from the minimal and maximal admittances of the AlN/Si bilayer LVRs with a thickness ratio of 10, N = 2 and DF = 0.8. The *K*^2^ is then calculated, as illustrated in Figure 4. The *K*^2^ of the IDT-grounded configuration is roughly half that of the IDT-floating configuration. Additionally, at certain normalized thicknesses, *K*^2^ undergoes a rapid decrease followed by a swift increase in both IDT-grounded and IDT-floating configurations when the second QS_0_ mode approaches even-order QA_0_ modes. This phenomenon of rapidly decreasing and then increasing of *K*^2^ is termed as a “degenerate valleys” in this article. However, only the IDT-grounded configuration shows a valley in *K*^2^ when the second QS_0_ mode intersects with odd-order QA_0_ modes, whereas the IDT-floating configuration does not. The valleys in *K*^2^ of AlN/Si bilayer LVRs are not observed in the *K*^2^ of the AlN/Si bilayer plate with Adler’s approach or the $k_{t}^{2}$ of AlN Lamb wave resonators [2].

**Figure 5:**
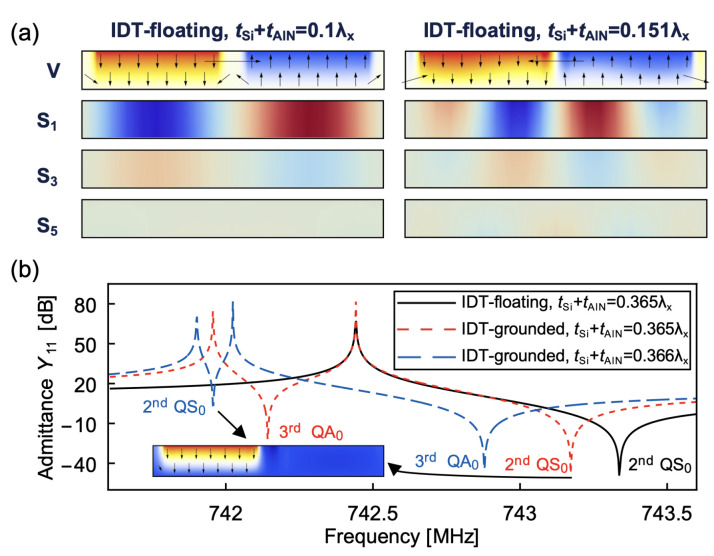
(**a**) Potential and strains of AlN/Si bilayer LVRs under IDT-floating configuration with the normalized thickness of 0.13 and 0.151, while the arrows indicate the electric fields; (**b**) admittance of AlN/Si bilayer LVRs when second QS_0_ and third QA_0_ modes are approaching.

The energy definition of *K*^2^ in (4) can be used to explain the above-mentioned phenomenon by examining the distribution of strains and electric field at $f_{p}$ [34]. Figure 5a displays the potential and strains distribution of the AlN layer in the LVR with the IDT-floating configuration. The bottom electrode of the AlN has roughly half the applied voltage on the IDT. In the regions between the Source Electrode–Bottom Electrode (SE–BE) and Ground Electrode–Bottom Electrode (GE–BE), the electric field is primarily $E_{3}$ [35]. At a normalized thickness of 0.1, the predominant strains are $S_{1}$ and $S_{3}$. $S_{1}$ is characterized by tension in the SE–BE region and compression in the GE–BE region, whereas $S_{3}$ exhibits the opposite behavior. As the second QS_0_ mode and fourth QA_0_ mode approach (e.g., at a normalized thickness of 0.151), an increased level of bending is observed in the mode shape, leading to a mixture of both tensile and compressive strains in both the SE–BE and GE–BE regions, whereas the potential distribution remains unchanged. This leads to a reduction in $W_{\mathrm{mutual}}$ in (4) and a corresponding decrease in *K*^2^. In the IDT-grounded configuration, the electric field is double in the SE–BE region but is absent in the GE–BE region. Therefore, the valley in *K*^2^ also occurs when the second QS_0_ mode and even-order QA_0_ modes approach. In this configuration, the integral regions of $W_{\mathrm{mutual}}$ and $W_{\mathrm{mech}}$ are only the SE–BE region, which is half that of the IDT-floating configuration, resulting in the $k_{t}^{2}$ of IDT-grounded being approximately half that of IDT-floating.

The IDT-floating configuration eliminates the excitation of odd-order QA_0_ modes due to the asymmetric electric field and symmetric strains to $x_{1}$. However, the IDT-grounded configuration allows for the excitation of these modes as the electric field is present only in the SE–BE region. When the second QS_0_ mode is excited simultaneously with the third QA_0_ spurious mode, the extracted *K*^2^ value from $f_{p}$ and $f_{s}$ becomes inaccurate. To obtain the precise *K*^2^ of the second QS_0_ mode, it is necessary to fit the admittance using two motional branches [33]. Consequently, only the cases where the second QS_0_ mode approaches even-order QA_0_ modes hold significance, and the subsequent sections will only focus on the IDT-floating configuration.

## 4. Effect of Structural Parameters on *K*^2^ and Valleys

In the previous discussions, the thickness ratio is set to 10 and NF = 2, DF = 0.8, but these parameters significantly impact the *K*^2^ of AlN/Si bilayer LVRs. Hence, in the forthcoming analysis, the effect of varying NF, thickness ratio and DF on *K*^2^ and their impacts on the valleys of *K*^2^ will be thoroughly analyzed.

**Figure 6:**
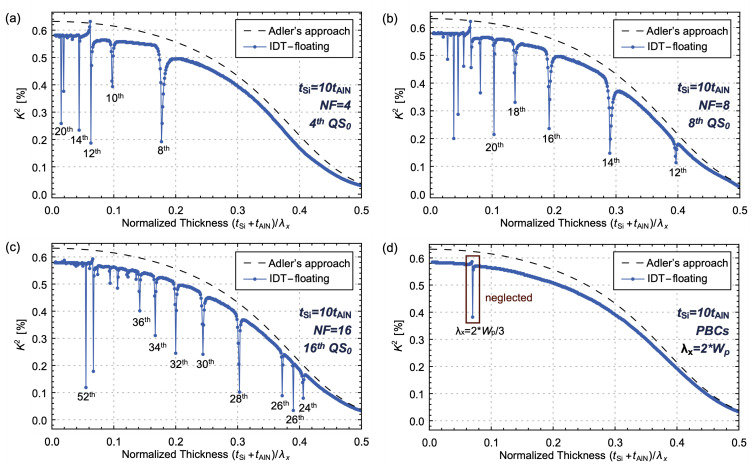
The simulated *K*^2^ of AlN/Si bilayer LVRs with (**a**) NF = 4, (**b**) NF = 8, (**c**) NF = 16 and (**d**) PBCs. The thickness ratio is set to 10 and DF = 0.8.

The influence of the parameter NF on the *K*^2^ of AlN/Si bilayer LVRs, which operate in the NF-order QS_0_ mode, is firstly analyzed. The *K*^2^ of TPoS LVRs may exhibit valleys around the normalized thicknesses when the resonant frequency of the higher even-order QA_0_ modes and the QS_0_ mode approach. As depicted in Figure 4, the valleys of *K*^2^ in the IDT-grounded configuration are caused by the second QS_0_ mode approaching the fourth, sixth, eighth and tenth QA_0_ modes. Figure 6 shows that, as NF increases, the order of QA_0_ modes also increases and the rightmost valleys of *K*^2^ arise from the eighth, twelfth and twenty-fourth QA_0_ modes for NF = 4, 8 and 16, respectively. Furthermore, the phase velocity dispersion curves are closer for higher-order QA_0_ modes, resulting in a smaller distance between the adjacent valleys of *K*^2^ and more valleys are observed for a higher NFs.

As the NF increases, the curve of *K*^2^ with the change in normalized thickness gradually converges. Therefore, PBCs are utilized to simulate TPoS LVRs with infinite NFs, as depicted in Figure 6d. The IDT period length (double of center-to-center electrode pitch $W_{p}$) is an integer multiple of the wavelength of its resonant mode, $2W_{p}=n\times \lambda_{x}$, and the operating wavelength of the QS_0_ mode is $\lambda_{x}=2W_{p}$. One valley of *K*^2^ for the IDT-floating configuration is shown in Figure 6d. When the normalized thickness is around 0.7, both the QS_0_ mode with wavelength $2W_{p}$ and the QA_0_ mode with wavelength $2W_{p}/3$ can be excited near resonant frequency, which induces the valley of *K*^2^. Similarly, the valley arises from the spurious mode and should be neglected. Therefore, for bilayer LVRs with high NF, the *K*^2^ evaluated from (10) has the same trend as Adler’s approach, whereas little difference exists owing to the mismatch.

**Figure 7:**
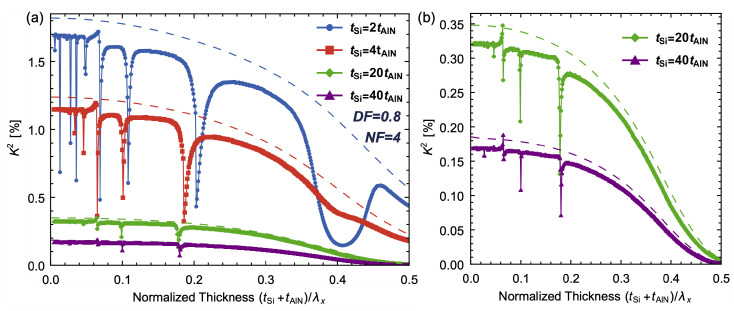
(**a**) The simulated *K*^2^ of AlN/Si bilayer LVRs with thickness ratios of 2, 4, 20 and 40, with NF = 4 and DF = 0.8; (**b**) magnified view of *K*^2^ with thickness ratios of 20 and 40.

The impact of the thickness ratio is analyzed through simulations of AlN/Si bilayer LVRs with different thickness ratios of 2, 4, 20 and 40 while fixing NF and DF at 4 and 0.8, respectively. The results depicted in Figure 7 show that an increase in the thickness ratio causes a decrease in *K*^2^, as previously noted. As the thickness ratio approaches 1, the mismatch between the AlN/Si bilayer structure becomes more pronounced, leading to wider valleys of *K*^2^, as shown in Figure 7. For the thickness ratio of 40, the valleys become very narrow, and fewer valleys are visible due to the limited resolution of simulation at a lower normalized thickness. Nevertheless, the order of QA_0_ modes remains unchanged, which is primarily determined by the NFs. Additionally, the deviation between *K*^2^ obtained from (10) and Adler’s approach decreases as the thickness ratio increases. With a thickness ratio of 2, a U-shape trend can be observed around the normalized thickness of 0.4, caused by the secondary proximity of the fourth QS_0_ branch and eighth QA_0_ branch with the modal transition, which impacts the shape of the QS_0_ mode.

**Figure 8:**
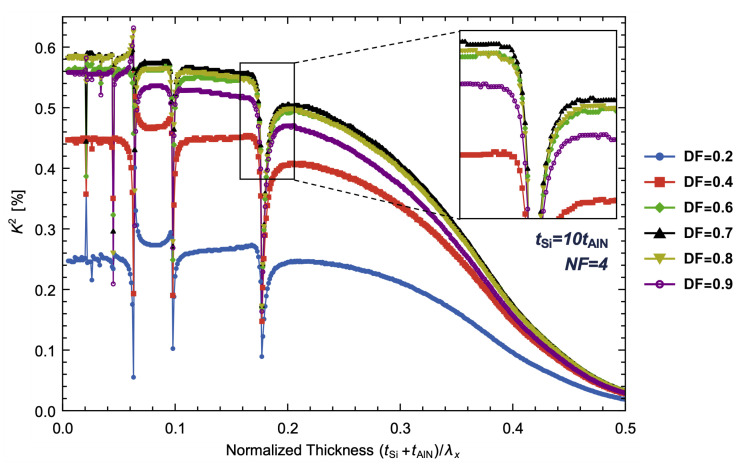
The simulated *K*^2^ of AlN/Si bilayer LVRs with DF = 0.2, 0.4, 0.6, 0.7, 0.8 and 0.9. The thickness ratio is set to 10 and NF = 4.

The AlN/Si bilayer LVRs with DFs of 0.2, 0.4, 0.6, 0.7, 0.8 and 0.9 are analyzed and the corresponding *K*^2^ are displayed in Figure 8. The effect of covering the electrode on the resonant mode of TPoS LVRs is limited to its boundary conditions, specifically the equipotential surface or normal electric displacement being zero, which has a minimal impact on the calculation of the resonant mode. Figure 8 demonstrates that a variation in the DFs has a negligible effect on the normalized thickness and width of the valleys. However, it does impact the potential distribution of the AlN layer. As the DFs increase from 0.2 to 0.7, the electric field is primarily $E_{3}$, leading to an increase in *K*^2^ due to the growth of the effective lateral area of the electric field, as described in (4). As the DFs continue to increase, $W_{\mathrm{elec}}$ increases but the displacements at the node is almost zero, resulting in a minimal change in $W_{\mathrm{mutual}}$, causing *K*^2^ to begin to decrease. This trend is confirmed by comparing DF = 0.7 to DF = 0.9, where *K*^2^ slightly decreases, as illustrated in Figure 8.

## 5. Conclusions

The integration of a structural layer within the piezoelectric bilayer plate reduces the phase velocity of the QS_0_ mode and *K*^2^. For piezoelectric bilayer LVRs, any two QL modes with the same parity order are not decoupled, resulting in a modal transition between the QS_0_ and QA_0_ modes when two QL branches approach. Notable degenerative valleys emerge due to the mismatch between electric and strain fields induced by modal transition. Altering structural parameters affects *K*^2^ and valleys as follows:

1. With an increase in the NF, the number of valleys rises while their width decreases. Additionally, the *K*^2^ trend corresponding to changes in normalized thickness converges.
2. As the thickness ratio approaches 1, *K*^2^ increases and the increase in the bilayer structure’s mismatch leads to an expansion in valley width. However, the number and position of the valleys remain unaffected.
3. The valleys are almost unaffected by NF, and *K*^2^ typically displays a pattern of initial increase followed by a decline as the DFs increase.

When designing piezoelectric bilayer LVRs, it is crucial to minimize *K*^2^ reduction by placing the normalized thickness away from the valleys, especially for bilayer LVRs with low thickness ratios. The observations and conclusions concerning valleys in the AlN/Si bilayer LVRs can also serve as a reference for other LVRs such as LiNbO_3_/SiO_2_ for temperature compensation.

## Figures and Tables

**Table 1:** Material constants of AlN and Si used in the simulations.

	Symbol	AlN	Si	Unit
Elastic constants	$c_{11}$	345	166	$10^{9}\;N/m^{2}$
	$c_{13}$	120	64	
	$c_{33}$	395	166	
	$c_{44}$	118	80	
Mass density	ρ	3260	2330	$\mathrm{kg}/m^{3}$
Relative dielectric constants	$\varepsilon_{11}/\varepsilon_{0}$	9.0	/	1
	$\varepsilon_{33}/\varepsilon_{0}$	10.7	/	
Piezoelectric constants	$e_{15}$	−0.48	/	1
	$e_{31}$	−0.58	/	
	$e_{33}$	1.55	/	

**Table 2:** Different definitions of *K*^2^ in this article.

Equation	Description
(2)	Adler’s approach
(3)	energy definition, general definition for different types of resonators
(4)	energy-definition-based two-dimensional definition under the plane strain assumption
(5)	energy-definition-based one-dimensional approximation for AlN/Si bilayer LVR under the electrode configuration of IDT-floating
(7)	energy-definition-based one-dimensional approximation for AlN/Si bilayer LVR under the electrode configuration of IDT-grounded
(9)	Adler’s-approach-based one-dimensional approximation for AlN/Si bilayer LVR under the electrode configuration of IDT-floating or IDT-grounded
(10)	resonant-frequencies-based definition, commonly used in experiments for simplification

## Data Availability

The data presented in this study are available upon request from the corresponding author.
